# Hair-Reinforced Elastomer Matrix Composites: Formulation, Mechanical Testing, and Advanced Microstructural Characterization

**DOI:** 10.3390/polym15224448

**Published:** 2023-11-17

**Authors:** Eugene S. Statnik, Julijana Cvjetinovic, Semen D. Ignatyev, Loujain Wassouf, Alexey I. Salimon, Alexander M. Korsunsky

**Affiliations:** 1 «LUCh» Lab, NUST MISIS, 119049 Moscow, Russia; 2 Center for Photonic Science and Engineering, Skoltech, 121205 Moscow, Russia; 3 Department of Physical Chemistry, NUST MISIS, 119049 Moscow, Russia; 4 Trinity College, University of Oxford, Broad St., Oxford OX1 3BH, UK

**Keywords:** hair, polymer, composite, carbon, fracture, SEM

## Abstract

Epoxy matrix composites reinforced with high-performance fibers, such as carbon, Kevlar, and glass, exhibit excellent specific stiffness and strength in many mechanical applications. However, these composites are disappointingly non-recyclable and are usually disposed of in landfill sites, with no realistic prospect for biodegradation in a reasonable time. In contrast, moldable composites with carbonized elastomeric matrices developed in the last decades possess attractive mechanical properties in final net-shape products and can also be incinerated or recycled. Many carbon and inorganic fillers have recently been evaluated to adjust the properties of carbonized elastomeric composites. Renewable organic fillers, such as human or animal hair, offer an attractive fibrous material with substantial potential for reinforcing composites with elastomeric matrices. Samples of unidirectional fiber composites (with hair volume fractions up to 7%) and quasi-isotropic short fiber composites (with hair volume fractions up to 20%) of human hair-reinforced nitrile butadiene rubbers (HH-NBRs) were produced in the peroxide-cured and carbonized states. The samples were characterized using scanning electron microscopy (SEM), Raman spectroscopy, and photoacoustic microscopy. Mechanical tests were performed under tension using a miniature universal testing machine. The expected effect of fiber reinforcement on the overall mechanical performance was demonstrated for both cured and carbonized composites. Considerable enhancement of the elastic modulus (up to ten times), ultimate tensile strength (up to three times), and damage tolerance was achieved. The evidence of satisfactory interfacial bonding between hair and rubber was confirmed via SEM imaging of fracture surfaces. The suitability of photoacoustic microscopy was assessed for 3D reconstructions of the fiber sub-system’s spatial distribution and non-destructive testing.

## 1. Introduction

Hair is a readily available, affordable, renewable, and durable biopolymer material that is well known and traditionally used by humans for fabrication and construction since the Neolithic epoch [1]. Human hairs and animal bristles have been used for diverse house appliances (e.g., brushes and polishing tools), construction materials, bijouterie, clothing, musical instruments, and, more specifically, in mechanical applications to reinforce technical fabrics and insulating mats [2], clay [3], brick, and concrete [4].

**Figure 1:**
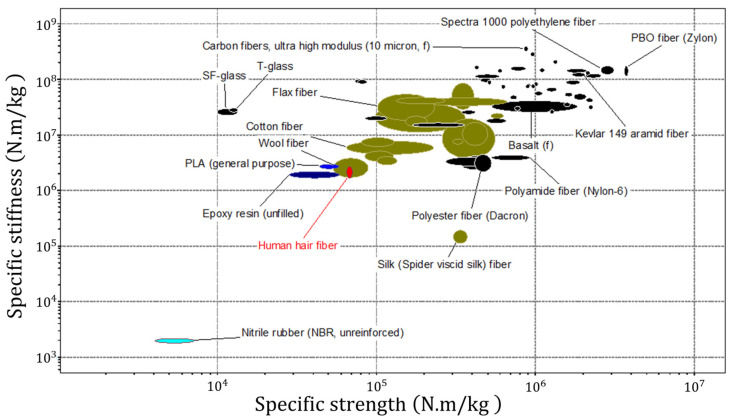
Ashby’s chart for the mechanical performance of fiber materials. This image was generated using Ansys Granta Selector 2021 R1 software [7].

Recently, human hairs have been evaluated as reinforcing fibers in epoxy composites [5] and 3D-printable PLA [6]. Taking into account that hairs do not exhibit outstanding specific stiffness and strength when compared to many other fibrous materials, as shown in Figure 1, it is necessary to provide strong arguments to justify their use for polymer matrix reinforcement in engineering composites.

We formulated such arguments as follows:

- *Eco-friendliness*: Since the hairs are bioderived fibers based on the natural polymer keratin, they represent a natural, cheap, renewable, and biodegradable alternative to polyester, glass, and carbon fibers. However, it should also be noted that cellulose-based fibers derived from plants, such as flax and cotton, can be readily produced on an industrial scale and generally exhibit superior mechanical property reproducibility.
- *Attractive surface structure*: Hair is a complex, hierarchically structured object with an intricate internal surface [8] structure, which has been extensively studied using various microscopy and tomography methods over decades [9]. Keratin scales on the surface of hair fibers are considered to be effective mechanical hooks for providing a strong interface bond with the matrix, which can be further improved through chemical modification (activation) [10,11].
- *Rational choice of matrix material*: Hairs are very flexible in bending and under tension show large values of elongation to rupture of up to 50%. The specific strength and stiffness of hairs are almost identical to epoxy and PLA, indicating that little or no reinforcement effect can be anticipated in composites using these matrices. The overall mechanical performance of hairs (low stiffness and good flexibility) indicates that the reinforcement effect is likely to be more pronounced for polymer matrices with lower stiffness, such as elastomers [12,13].

In this article, we examined several aspects of hair fiber reinforcement for elastomer matrix composites. To the best of our knowledge, this article is the first report on human hair-nitrile butadiene rubber (HH-NBR) composites that have been intentionally formulated, fabricated, characterized, and tested to investigate the potential of this class of engineering materials.

NB rubber compounds have various applications in the oil and gas, chemical, automotive, and general engineering industries due to their excellent combination of mechanical performance, chemical stability, water resistance, and solvent resistance, as well as temperature resistance. Various inorganic fillers, such as carbon black, graphite, silica, etc., along with curing and processing agents are added to the NB *caoutchouc* by industrial producers to tailor the properties of the vulcanized compound and to match the requirements posed by the specific service conditions.

In the last decade, a specific class of carbon-based composites known as carbonized elastomeric composites have been developed [14]. These composites aim to combine the benefits of traditional rubber technology, such as high filling ratios and precise molding, with gentle low-temperature carbonization, ultimately targeting the mass production of high-quality engineering parts with high mechanical performance [15]. This class of materials is a competitor of magnesium and aluminum alloys in terms of mechanical performance but offers advantages in chemical and temperature resistance [16].

Composite and hybrid materials with biopolymer matrices or fillers are crucial for promoting sustainable development through the use of renewable resources as raw materials. The significance of these materials cannot be overstated. Creating such materials encounters common difficulties observed in composite materials, such as the requirement for optimal spatial arrangement of reinforcing structural elements, improved adhesion between the filler and the matrix, and quality control of finished products via non-destructive methods.

In this study, we fabricated unidirectional and quasi-isotropic short fiber composites by introducing human hair reinforcements (without any other fillers) into a peroxide-curable NB *caoutchouc* (natural isoprene) matrix. Plates of composites were thermally pressed to vulcanize the NB matrix and to prepare elastomers for subsequent carbonization. The vulcanized and carbonized composites were characterized using SEM, Raman spectroscopy, and photoacoustic microscopy. The latter technique utilizes the photoacoustic effect of melanin present in hairs as a natural pigment. This has been applied for the first time in composite material science to obtain a non-destructive visualization of the internal filler spatial distribution in 3D. We discussed the appearance of fracture surfaces in samples after tensile mechanical testing in connection with a strong reinforcement effect caused by hairs in both vulcanized and carbonized composites.

## 2. Materials and Methods

### 2.1. Samples Preparations

The nitrile butadiene caoutchouc BNKS-18 (Sibur Inc., Krasnoyarsk, Russia) was cut into pieces for softening and repeatedly rolled for 10 min using a laboratory two-roll rubber mill BL-6175-A (Dongguan Baopin Precision Instrument Co., Dongguan, China) to intermix caoutchouc with human hairs and dicumyl-peroxide (C_18_H_22_O_2_, CAS number 80-43-3, Sigma-Aldrich Corp., St. Louis, MO, USA). “Pristine” human hairs of average length of about 100 mm were donated in their natural state by one of authors to the total amount of 185 g. The human hair used has not previously undergone any chemical, thermal, or coloring treatments.

To produce unidirectional composites, a significant amount (up to 400 g) of rubber was rolled to a thickness of 1.5 mm. Long human hairs (approximately 8 g per 100 g of NB cauthchouque) were introduced into the inter-roll gap, manually guiding the hairs to run parallel to the rolling direction. The compound was mixed for 25 min at a temperature of approximately 80 °C. Then, the mixture was cooled down to 40 °C before introducing 3 g of the dicumyl-peroxide per 100 g of NB caoutchouc to produce rubber compounds. The duration of the final mixing stage was 10 min to ultimately obtain 1.2~1.4 mm-thick sheets of compound ready for vulcanization. This sheet was cut into 145 × 145 mm^2^ squares. Next, they were put into stacks of 6 slices, which allowed to create the 0/0/0/0/0/0 and 0/90/0/90/0/90 stacking sequences. The stacks were vulcanized under plate compression to 10 MPa pressure at 170 °C for 10 min in air using an automated hydraulic 40 tons press (Tesar Engineering Ltd., Saratov, Russia). Vulcanized sheets having thickness of about 3.5 mm were cut using a hardened steel punching tool to obtain dog-bone samples for tensile testing. Depending on the orientation of the punching tool, specimens with hair reinforcements oriented parallel (II) or perpendicular (=) to the gage axis were produced from 0/0/0/0/0/0 stacks. Additionally, specimens with hair reinforcements oriented both parallel and perpendicular were cut from 0/90/0/90/0/90 stacks after vulcanization. Due to the limited amount of long hairs from a particular person, only a few samples with the specified dimensions could be produced; therefore, statistical analysis was also limited in this aspect. Unfortunately, the attempts to increase the filling ratio for unidirectional composites also failed using the current technology, as long hairs tended to break into shorter pieces during mixing and lost their desired orientation.

Quasi-isotropic composites were fabricated similarly, i.e., long hairs were cut into segments, chopped down to 20 mm, and randomly introduced into a NB caoutchouc matrix, which was then freely poured onto the surface of another caoutchouc material entrapped between mill rolls. In contrast to unidirectional composites, the volume fraction of reinforcing fibers in the former could be increased by up to 20% to investigate the impact of a high filling ratio. Moreover, the use of miniature specimens for in-SEM mechanical testing allowed for improving statistics and increasing fidelity.

The overall approach to the composite sample preparation of carbon composites in this class has been thoroughly studied, validated, and reported by the current team of authors in previous publications. Further details can be found in the references [14,15,16].

### 2.2. Scanning Electron Microscopy

The fracture surfaces of both vulcanized and carbonized types of samples, as well as hair distribution and structure, were investigated using a versatile FIB-SEM Tescan Amber (TESCAN GROUP, Brno, Czech Republic) under the following conditions: SE detector, working distance of 6 mm, landing energy of 5 kV, beam current of 300 pA, and 2K image size with 8-bit depth. Prior to investigation, the samples were sputtered with a very thin layer of gold nanoparticles using a Quorum Q150R ES Magnetron machine (Quorum Technologies, Judges Scientific, Lewes, UK).

### 2.3. Tensile Test

**Figure 2:**
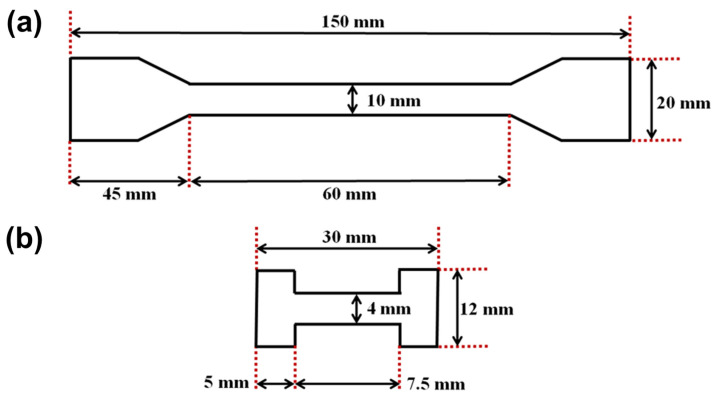
Sketches of the (**a**) large and (**b**) miniature samples.

Mechanical tests of the produced samples were conducted using a Zwick/Roell Z020 (Zwick GmbH, Ulm, Germany) universal testing machine equipped with a MultiXtens high-precision strain measuring system for large samples and a Deben MT 1 kN tensile tester (Deben UK Ltd., Woolpit, UK) for miniature samples, respectively. The sketches of the typical size and shape of the specimens are presented in Figure 2. In this case, ISO 37: “Rubber, vulcanized or thermoplastic—determination of tensile stress-strain properties” was used as a conceptual guide in terms of specimen shape and general procedure [17].

The large specimens were tested at a constant crosshead speed of 2 mm/min, while the miniature ones were examined at half that rate. The tests were carried out in an open-air environment and recorded using a digital camera. Subsequently, the resulting videos were processed with the free trial version of the commercial GOM Correlate Pro Digital Image Correlation (DIC) software (Zeiss INSPECT 2023, Carl Zeiss GOM Metrology GmbH, Braunschweig, Germany) to determine the true strains within the samples.

**Figure 3:**
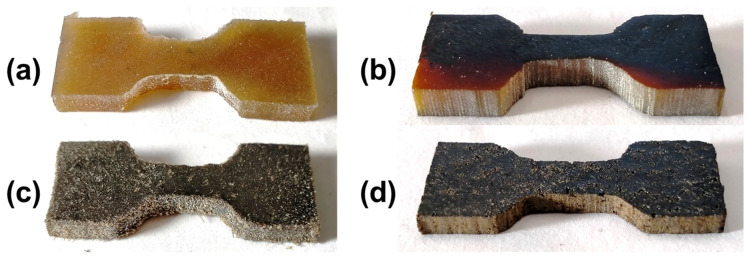
The appearance of the prepared miniature samples: (**a**) before carbonization without hairs, (**b**) after carbonization without hairs, (**c**) before carbonization with hairs, and (**d**) after carbonization with hairs.

The number of samples with different filling ratios and orientations was limited to 2 due to technological constraints. The proposed method made it challenging to align and reinforce larger samples. In contrast, 5 miniature specimens were employed to examine data dispersion. In this case, to localize deformation at the center of the specimen, small incisions were made on both sides perpendicular to the axis of tension with a depth of 0.5 mm and a width of 0.2 mm. The various miniature samples prepared using this method are shown in Figure 3.

### 2.4. Photoacoustic Microscopy

The raster-scanning optoacoustic mesoscopy system RSOM Explorer P50 (iTheraMedical GmbH, Munich, Germany) was employed to acquire photoacoustic signals from hair-reinforced elastomer matrix composites as well as raw elastomer matrices. Prior to imaging, the samples were placed in a plastic container and submerged in deionized water to ensure adequate ultrasound coupling with the transducer. Optical excitation was achieved using a Wedge-HB frequency-doubled flashlamp-pumped Nd:YAG laser (1064/532 nm wavelength; 1 ns pulse duration; 200 μJ pulse energy; and 1 kHz repetition rate) delivered through a 2-arm glass fiber bundle, resulting in a spot size ranging from 3.5 mm to 5.0 mm [18]. The scanning was performed in the field of view up to 12 × 12 mm^2^, with depths of up to 3 mm and a step size of 20 μm. To detect the induced ultrasound signals, a custom-made spherically focused LiNbO_3_ detector, with parameters including a center frequency of 50 MHz, a bandwidth of 11–99 MHz, a focal diameter of 3 mm, and a focal distance of 3 mm, was utilized. The RSOM setup provided axial and lateral resolution capabilities of 10 μm and 40 μm, respectively. The resulting signals were displayed in frequency ranges of 11–33 MHz (shown in red) and 11–99 MHz (merged).

## 3. Results and Discussion

### 3.1. Microstructure Characterization via Scanning Electron Microscopy

**Figure 4:**
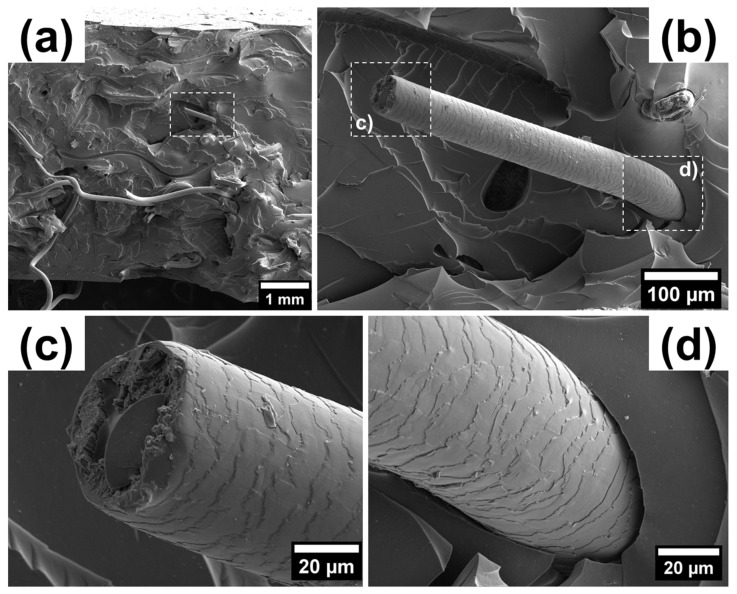
The fracture surface of a BN rubber-based vulcanized composite with a 7 vol.% hair reinforcement: (**a**) general view with indication of a specific protruding hair from the polymer matrix in the white dashed rectangular box, (**b**) detailed view of the hair structure with two selected regions like (**c**) the fracture zone and (**d**) the interface between the hair and the matrix.

**Figure 5:**
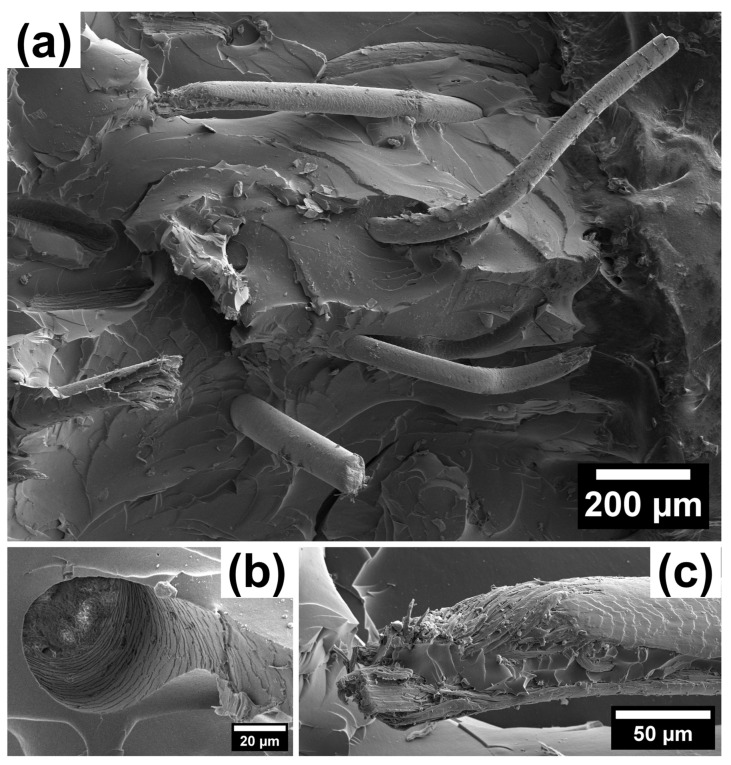
The counterpart of the fracture surface demonstrated in Figure 4: (**a**) large overview; (**b**) imprint of hair structure at the lacuna (matrix) periphery with the visible cross-section of fractured hair at the bottom (first fracture mechanism); and (**c**) appearance of hair structure after removal from polymer matrix (second fracture mechanism).

SEM images of a BN rubber-based vulcanized composite with a 7 vol.% hair reinforcement (in a mixed orientation with respect to the tension axis) taken at different magnifications are shown in Figure 4 and Figure 5. The fracture surface exhibits intricate relief with flat regions in the matrix, alongside many steps and well-preserved reinforcing hairs with characteristic ‘scale-like’ surface features. The presence of smooth facets and step features indicated the prevalence of the brittle failure mechanism. No micro-cracks were evident, but single tears were present, as shown in Figure 4a.

To assess the adhesion between hairs and matrix in the composites, SEM images of the hair surface morphology and matrix channels were analyzed. Several images revealed the characteristic imprints of hair surface morphology on the matrix after removal (such as tearing or loss of cohesion), indicating satisfactory adhesion. This suggests the establishment of a strong mechanical bond between the human hair fibers and the matrix, as illustrated in Figure 4b,d.

The pattern of the hair–matrix interface and structure of the fractured hairs shown in Figure 5b and Figure 5c, respectively, indicate that hair breaks under tension in a brittle manner, resulting in a relatively flat area on the fracture zone across the entire cross-section of the hair and a precise imprint of the hair structure in the matrix. Another fracture mechanism was also observed, in which a portion of the matrix material tears out, along with the hair undergoing plastic flow and ductile fracture due to sufficient hair–matrix adhesion. This mechanism can be observed in Figure 5c.

The hair surface is not smooth but displays a scale-like nature that improves the cohesion through mechanical ‘keying in’ within the matrix. The surface of human hair is naturally scaly. The small scales located at the hair surface create tiny steps or wedges. When the fibers are mixed with rubber, the rubber molecules penetrate these wedges, enhancing the physical interaction. This interaction between human hair and rubber improves the overall physical and mechanical properties. Some of the characteristics of human hair fibers that make them a promising reinforcement and strengthening material are their excellent tensile strength, slow degradation rate, hydrophilic properties, affordability, distinctive chemical composition, and elastic recovery capability.

**Figure 6:**
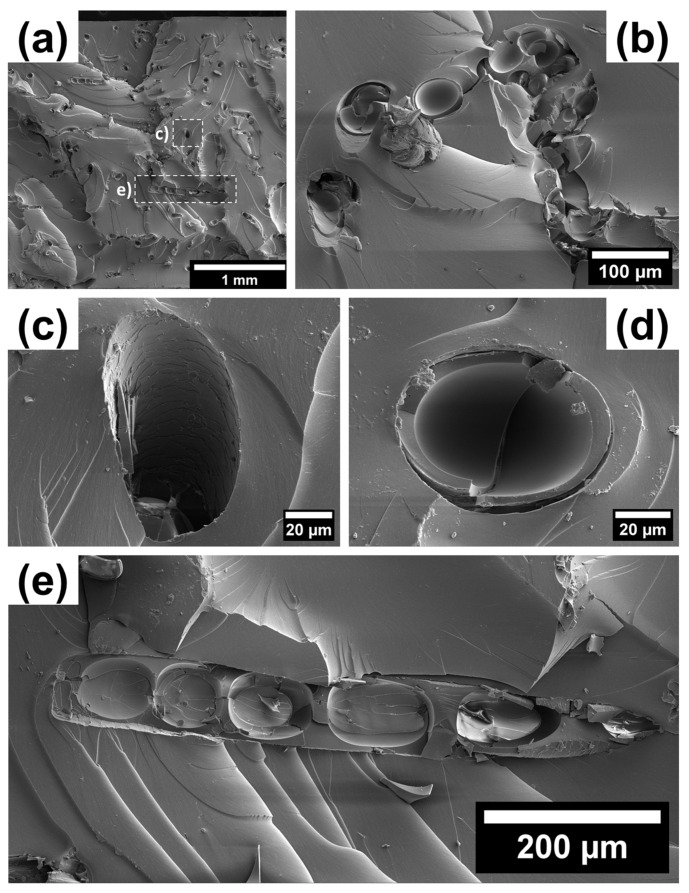
The fracture surface of a BN rubber-based carbonized composite with a 7 vol.% hair reinforcement: (**a**) general view with the indication of a (**b**) cross-section of the hairs conglomerate accumulated in the matrix due to poor mixing, (**c**) lacuna received after the tension test, (**d**) cross-sectional view of the hair structure, and (**e**) longitudinal slice of the fractured hair.

The fracture structure of carbonized composites with hair reinforcement based on BN rubber shown in Figure 6 differs from the vulcanized one due to its increased smoothness, namely with larger flat facets, lack of protruding free ends of reinforcing hairs from the matrix, and the formation of pores in empty channels following hair pullout.

The reinforcing fibers may display an inhomogeneous distribution throughout the matrix. The fracture of the fiber takes the shape of a smooth, concave rounded area with a set of cracks running parallel to the hair axis. This effect may be linked to thermal degradation. The cracks may propagate into the matrix, leading to the conclusion that carbonized hairs are no longer useful as effective crack blockers. The carbonization process results in a loss of the hair’s connectivity with the matrix, at least for a part of the reinforcing hair. Consequently, the hair–matrix interface is apparently weakened, making it easier for the reinforcing component to be pulled out from the matrix.

Based on the obtained views of the hair–composite microstructure, several approaches can be recommended to enhance the mechanical properties of vulcanized and carbonized elastomer composites reinforced with the addition of hair fibers, including:

- The optimization of the carbonization temperature to prevent the hair biopolymer’s thermal destruction;
- The chemical modification of the hair surface to enhance its adhesion to the elastomeric matrix, homogenizing the composite during rubber mixture preparation.

### 3.2. Mechanical Property Measurements

**Figure 7:**
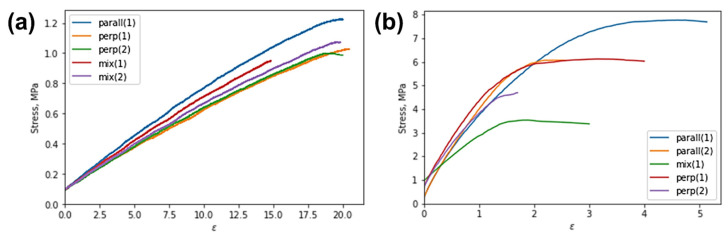
Engineering stress–strain curves obtained for large samples in tension: (**a**) vulcanized samples before carbonization; and (**b**) carbonized samples.

The stress–strain curves obtained for the large samples are shown in Figure 7. The pre-loading procedure was executed prior to the test to eliminate the occurrence of sample displacement within grips, which is why these curves do not begin at the zero point. The correspondent mechanical characteristics are estimated and summarized in Table 1.

There was a distinct correlation between Young’s modulus and the orientation of reinforcing hairs relative to the tension axis. Specifically, vulcanized samples with reinforcing hairs parallel to the tension axis have an elastic modulus that is 40% higher than those with perpendicular or mixed orientations. This phenomenon aligns with the rule-of-mixtures corresponding to the Voigt and Reuss models. In contrast, the orientation of hairs does not impact the ultimate strength and elongation to rupture. The values were distributed within a narrow range from 1.0 MPa to 1.2 MPa and from 15% to 23%, respectively.

The ultimate strength of carbonized specimens reached a maximum of 7.8 MPa when the reinforcing hair fibers were parallel to the tension axis. At the same time, samples with a mixed orientation of fibers had the lowest elongation to rupture, with a value of 0.02%. The elastic modulus of the carbonized composites do not show a clear dependence on fiber orientation.

On the other hand, carbonization of the vulcanized specimens entailed a noticeable increase in Young’s modulus and ultimate strength in general. For instance, the elastic modulus increase varied from 200% to 350% (when the reinforcing hairs were oriented parallel to the tension axis), from 400% to 500% (when the reinforcing hairs were oriented perpendicular to the tension axis), and increased by up to 250% (when the reinforcing hairs were placed in a mixed manner). Moreover, the ultimate strength increased by a factor ranging from 3.5 to 8 when compared to vulcanized samples.

It is worth noting that the carbonization process causes a significant embrittlement of the composite. In particular, when the reinforcing hairs were oriented parallel to the tension axis, there was a decrease in elongation to rupture of more than four times. Similarly, for samples with the hair orientation perpendicular to the tension axis, the drop was more than seven times. Finally, for samples with the mixed hair orientation distribution, the reduction in elongation to rupture for about three times was observed. The reduction in deformability (elongation to rupture) of the carbonized composites compared to the vulcanized samples was primarily attributed to the formation of a structure with a high carbon content (C-C) incorporating a small proportion of chemical bonds, such as C-H, N-H, and C-OH, as well as an increase in volume porosity.

**Figure 8:**
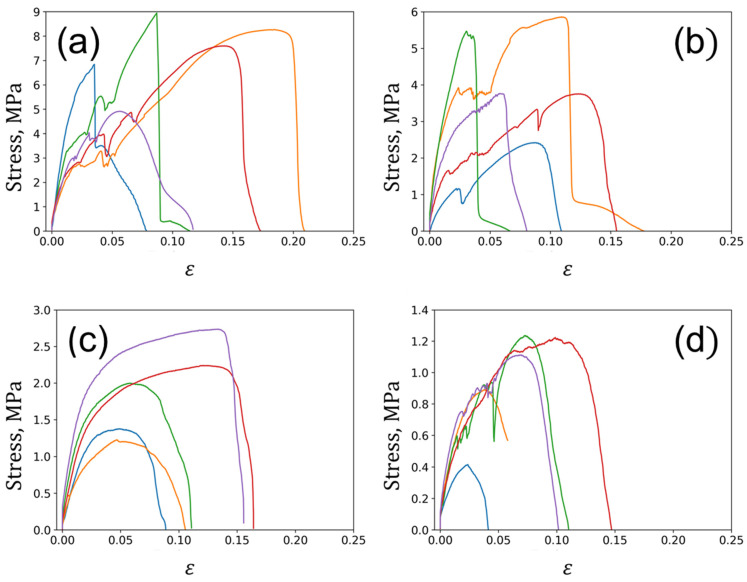
The stress–strain curves obtained for vulcanized miniature samples under tension: (**a**) without fillers; (**b**) without fillers with notches; (**c**) with hair reinforcement; and (**d**) with hair reinforcement and notches.

**Figure 9:**
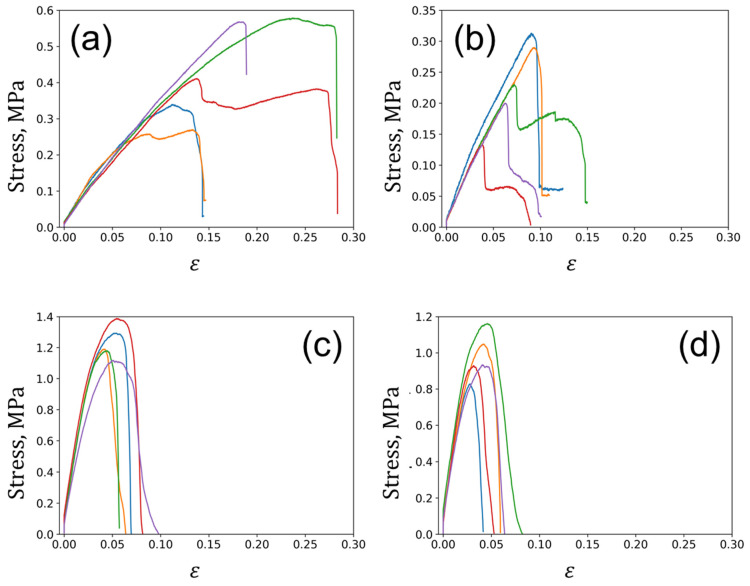
The stress–strain curves obtained for carbonized miniature samples in tension mode: (**a**) without fillers; (**b**) without fillers with notches; (**c**) with hairs reinforcement; and (**d**) with hairs reinforcement and notches.

The stress–strain curves obtained for the miniature samples are shown in Figure 8 and Figure 9. Their corresponding mechanical characteristics are estimated and summarized in Table 2.

The analysis of miniature samples provides more robust results from a statistical point of view due to the higher number of specimens. In this case, hair reinforcement results in a more than ten-fold increase from 3.7–3.8 MPa to 40 MPa in Young’s modulus for the composite materials in the vulcanized state compared to raw elastomeric materials.

The unfilled composite exhibited an increase of over seven times in the elastic modulus due to its carbonization. On the other hand, reinforced composites showed a somewhat lower increase—2.5 times for unnotched specimens and a modest rise of no more than 20% for notched specimens. The growth in Young’s modulus caused by matrix carbonization (as we believe) is weakened by the factors of porosity that form due to the thermal degradation of both the rubber matrix and the reinforcing hair biopolymer. Additionally, the introduction of a notch moderately decreases the effective elastic modulus in the unfilled composite and significantly reduces it by almost two times in the reinforced composite. The changes in tensile strength in the studied composite materials were similar to the variations in elastic modulus. The reinforcement of the elastomer matrix with human hairs gives a rise in tensile strength of about three times for both the notched and unnotched vulcanized samples. The notches themselves, however, reduce the effective strength, as commonly expected. Carbonization of vulcanized composites results in a more significant strength increase, by a factor of 18, for unreinforced specimens than reinforced ones, similarly to elastic modulus.

To sum up, hair reinforcement and notching in vulcanized composites reduce the overall deformability (elongation to rupture), while carbonization tends to improve it by 60% in reinforced samples. It is assumed that the cohesive porosity effectively eliminates cracks at the pores or redirects them away from the shortest propagation paths. Furthermore, the reinforcement significantly enhances the durability of composites, effectively blocking cracks and deviating them from the straight path.

The total work to failure of composites exhibits several distinctive features:

(a) An obvious decrease in this value in notched specimens compared to unnotched specimens (two and more times);
(b) An increase in the work of fracture resulting from carbonization (3–4 times);
(c) Unfavorable impact of reinforcement on the work of fracture (presumably caused by a decrease in deformability and effective cross-section).

### 3.3. Microstructure Characterization via Photoacoustic Microscopy

The idea was to use the melanin pigment as a sensitive marker to reveal hairs. In the article [19,20], the researchers investigated skin melanin using RSOM in the frequency range of 10–120 MHz as a proof of principle.

**Figure 10:**
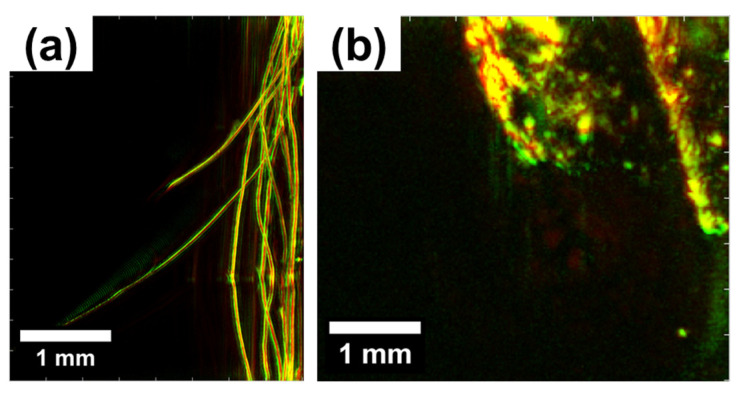
The photoacoustic signal obtained for (**a**) human hairs in the XZ plane and (**b**) unfilled elastomer material in the XY plane at the frequency range from 11 MHz to 99 MHz. The images are color-coded to represent the two reconstructed frequency bands (red: larger structures in the bandwidth of 11–33 MHz; green: smaller structures in the bandwidth of 33–99 MHz).

**Figure 11:**
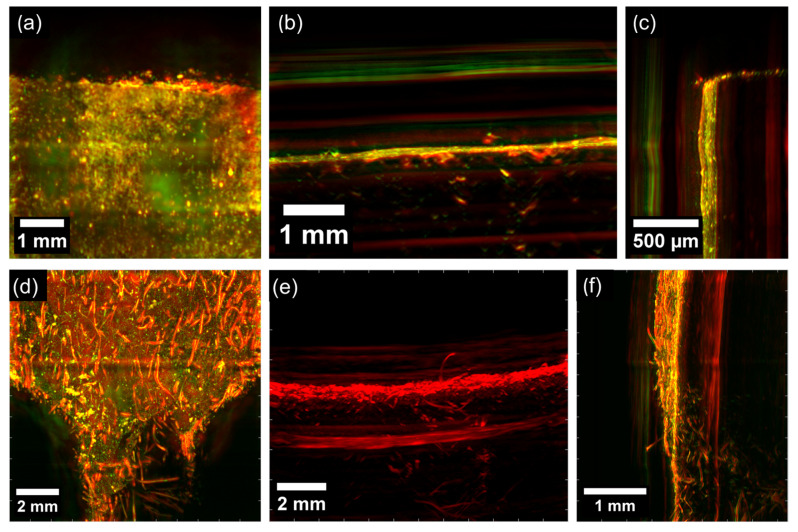
The photoacoustic signal obtained for (**a**–**c**) unfilled and (**d**–**f**) reinforced vulcanized composites at the frequency range from 11 MHz to 99 MHz (**a**–**d**,**f**) and 11–33 MHz (**e**). (**a**,**d**) The XY plane; (**b**,**e**) the YZ plane; (**c**,**f**) the XZ plane. The images are color-coded to represent the two reconstructed frequency bands (red: larger structures in the bandwidth of 11–33 MHz; green: smaller structures in the bandwidth of 33–99 MHz).

Photoacoustic images of human hairs and raw elastomer matrix are shown in Figure 10. The colors in the images correspond to the acoustic signals acquired at a frequency range of 11–99 MHz. The photoacoustic signal of the unfilled and reinforced rubber composite taken at different projections is depicted in Figure 11. The findings demonstrate that the reliable identification of reinforcing hairs can be achieved with this technique, providing good spatial resolution up to several microns. This confirms the random orientation and uniform distribution of hair throughout the matrix. The rubber matrix, as it is, generates a diffuse background signal, perhaps, due to surface refraction effects. When a laser beam is incident at the rubber–air interface, some of the light is reflected, some is absorbed, and some is transmitted into the rubber material. The absorbed light generates a photoacoustic signal due to the thermal expansion of the material and subsequent generation of ultrasound waves. In the case of rubber matrices, photons undergo multiple scattering events as they propagate through the material. These scattering events cause light to deviate from its original path and result in the diffusion of light within the rubber matrix. As a consequence, the generated photoacoustic signals also exhibit a diffuse nature, since the absorbed light is distributed throughout the volume of the matrix rather than being confined to specific absorption locations. However, photoacoustic microscopy fails to produce adequate acoustic signals to distinguish and recognize reinforcing hairs within carbonized samples. It is probable that the thermal destruction of hair biopolymers leads to the removal of photoactive melanin in the hairs.

These findings support the ability of a non-invasive visualization of reinforcing hairs within composite materials. This method can subsequently facilitate the 3D reconstruction of the material structure and the corresponding finite element analysis of the deformation behavior. Moreover, it offers the potential to transition to photoacoustic 3D tomography for composites and enable the non-destructive quality control of such materials. The use of markers in a polymer matrix provides opportunities for a variety of *operando* tests.

## 4. Conclusions

This study was focused on analyzing the structure and physical properties, as well as the mechanical properties, of composite materials. The materials were made of vulcanized and carbonized butadiene nitrile rubber and were strengthened with biopolymer fibers (human hairs).

From this study, the following inferences can be made:

- The feasibility of the proposed technique for producing composite materials that exhibit a marked increase in physical and mechanical properties (specifically elastic modulus and tensile strength), while maintaining acceptable levels of deformability and fracture work, has been verified in comparison to unreinforced rubber.
- The experimental results confirm a significant enhancement in properties for both vulcanized and carbonized NR matrix rubber under varying hair orientations (i.e., parallel, perpendicular, and mixed) relative to the tensile axis with and without notches (i.e., stress concentrators).
- Notably, the hair-reinforced composites display a unique fracture behavior that leads to high specimen survivability even after the onset of fracture.
- The study utilizes high-resolution scanning electron microscopy to analyze the fracture surface and proposes mechanisms for fracture development. Specifically, it identifies the structural characteristics of the matrix and reinforcing fibers that effectively block brittle crack propagation.
- The effectiveness of using nanostructured melanin as a marker for photoacoustic imaging of fibers within the matrix is also demonstrated. High-resolution images were acquired to observe the reinforcing fibers within the elastomeric matrix.

## Figures and Tables

**Table 1:** The mechanical properties of large samples obtained using a Zwick/Roell Z02 universal testing machine.

Composition	E (MPa)	σ_max (MPa)	Elongation till Rupture (%)
Vulcanized samples before carbonization			
parall(1)	10.0	1.2	23
perp(1)	5.7	1.0	22
perp(2)	6.6	1.0	21
mix(1)	6.7	1.0	15
mix(2)	6.1	1.1	21
Carbonized samples			
parall(1)	282	7.8	5
parall(2)	334	6.1	3
mix(1)	203	3.5	5
perp(1)	396	6.1	6
perp(2)	281	4.7	2

**Table 2:** The mechanical properties of miniature samples obtained using a Deben Microtest 1 kN universal testing machine.

	With Notches	With Hairs	Young’s Modulus (MPa)	Tensile Strength (MPa)	Elongation till Rupture (%)	Fracture Work (N·mm)
Vulcanized			3.7 ± 0.1	0.4 ± 0.1	21 ± 3	10 ± 2
	+		3.8 ± 0.2	0.23 ± 0.03	11 ± 1	2.2 ± 0.4
		+	42 ± 2	1.23 ± 0.04	7 ± 1	6 ± 1
	+	+	39 ± 1	1.0 ± 0.1	6 ± 1	4 ± 1
Carbonized			298 ± 27	7.3 ± 0.6	14 ± 2	81 ± 21
	+		257 ± 54	4.3 ± 0.6	12 ± 2	39 ± 10
		+	103 ± 16	1.9 ± 0.2	12 ± 1	19 ± 5
	+	+	53 ± 6	1.0 ± 0.1	9 ± 2	7 ± 2

## Data Availability

The data presented in this study are available on request from the corresponding author.
